# iTRAQ and PRM-based quantitative proteomics in early recurrent spontaneous abortion: biomarkers discovery

**DOI:** 10.1186/s12014-019-9256-y

**Published:** 2019-10-18

**Authors:** Ying Cui, Ling He, Chun-Yan Yang, Qian Ye

**Affiliations:** 1 Jiangxi Maternal and Child Health Hospital, Nanchang, 330006 China; 2 The Affiliated Hospital and Clinical Institute, Jiangxi University of Traditional Chinese Medicine, No. 445, Bayi Avenue, Donghu District, Nanchang, 330006 China; 3 Jiangxi University of Traditional Chinese Medicine, Nanchang, 330004 China

**Keywords:** Early recurrent spontaneous abortion, Proteomics, Biomarker

## Abstract

**Background:**

Early recurrent spontaneous abortion (ERSA) is a common condition in pregnant women. To prevent ERSA is necessary to look for abortion indicators, such as hormones and proteins, in an early stage.

**Methods:**

Thirty patients with ERSA were enrolled in the case group. In the control group, we recruited 30 healthy women without a history of miscarriage undergoing voluntary pregnancy termination. The differentially expressed proteins in the serum were identified between the two groups using PRM and iTRAQ.

**Results:**

Seventy-eight differentially expressed proteins were identified. Using GO functional annotation and KEGG pathway analysis, we detected that the most significant changes occurred in the pathway of Fc gamma R-mediated phagocytosis. Meanwhile, using PRM, we identified three proteins that were closely related to abortion, B4DTF1 (highly similar to PSG1), P11464 (PSG1), and B4DF70 (highly similar to Prdx-2). The levels of B4DTF1 and P11464 were down-regulated, while the level of B4DF70 was up-regulated.

**Conclusions:**

CD45, PSG1, and Prdx-2, were significantly dysregulated in the samples of ERSA and could become important biomarkers for the prediction and diagnosis of ERSA. Larger‑scale studies are required to confirm the diagnostic value of these biomarkers.

## Background

Early recurrent spontaneous abortion (ERSA), also called recurrent pregnancy loss (RPL), is a disease distinct from infertility, defined by two or more failed pregnancies [1]. According to the guidelines of the Royal College of Obstetricians and Gynecologists (RCOG), it is defined as a spontaneous abortion that occurs three times or more within the first 12 weeks of pregnancy and with the same sexual partner [2]. There are still many unknown causes of abortion besides those caused by genetics, autoimmune abnormalities, endocrine, anatomy, or pre-thrombotic state [2]. The incidence of ERSA is about 5% and advancing maternal age and history of multiple miscarriages are high-risk factors for ERSA [1, 3]. Approximately in half of the patients with RPL, there is no explanation for their miscarriages [4]. Therefore, early prediction of the potential risk of ERSA is needed to increase the live birth rates in patients with ERSA [5].

Proteomics is an emerging discipline which involves the global analysis of changes in protein expression [6]. The application of proteomics technology had a significant impact on the etiology and pathogenesis assessment of many diseases, especially cancer, cardiovascular disease, diabetes, and neurological disorders [7–9]. Klein et al. [10] pointed out that proteomics can be useful for prediction, diagnosis, management, monitoring, and prognosis of several obstetric conditions that are associated with an increased risk of maternal and/or perinatal morbidity and mortality.

Numerous proteomic studies have shown that the human proteome regulates cellular function and determines the phenotype; therefore, the identification of relevant proteins is likely to reveal reliable biomarkers for disease prediction [11]. Some potential biomarkers for ERSA have been previously reported. Previous studies using LC–MS/MS and ELISA showed a significant decrease in the levels of insulin‑like growth factor‑binding protein‑related protein 1 (IFGBP‑rp1)/IGFBP‑7, Dickkopf‑related protein 3, the receptor for advanced glycation end products (RAGE), and angiopoietin‑2 in patients with RSA [12]. Kim et al. [13] used blood samples from healthy and RPL patients to conduct a comparative proteomic study, they performed 2D-PAGE and the selected spots were analyzed with MALDI-TOF/MS. Their results suggested that inter-α trypsin inhibitor-heavy chain 4 (ITI-H4) expression might be used as a biomarker. Using isobaric tags for relative and absolute quantification (iTRAQ) and ingenuity pathway analysis (IPA), Pan et al. [14] observed some altered protein expression in the placental villous tissue of patients with early recurrent miscarriage.

Searching for new biomarkers of ERSA is helpful for diagnosis, safety, and efficacy evaluation of the disease. Advances in proteomics have made this effort more efficient. However, there is no previous study that identified serum RSA biomarkers using parallel reaction monitoring (PRM). Therefore, in this study, except for iTRAQ and bioinformatics analysis, such as protein–protein interaction (PPI) network analysis, GO and KEGG, we used PRM to identify reliable biomarkers for the prediction of RSA.

## Materials and methods

### Patients and controls

From October 2017 to December 2017, in the case group, we recruited 30 patients that had a previous abortion. Our inclusion criteria were based on the consensus of Practice Committee of the American Society for Reproductive Medicine (ASRM) and RCOG [1, 2]. All the patients were diagnosed as ERSA except for chromosomal abnormalities, anatomical abnormalities, endocrine diseases, anatomical abnormalities of the genital tract, infections, immunologic diseases, trauma, and internal diseases. Gestational sacs without fetal heart rate were found using transvaginal ultrasound.

Meanwhile, in the control group, we recruited 30 women who terminated their pregnancy and did not have a history of abortion. The inclusion criteria we the following: women who underwent pregnancy termination at a gestational age of 6–10 weeks and had no previous history of recurrent spontaneous abortions, chromosomal abnormalities, anatomical abnormalities, endocrine diseases, anatomical abnormalities of the genital tract, infections, immunologic diseases, trauma, internal diseases, or any chemical agent intake before their pregnancy terminations [15].

Characteristics of participants were summarized in Table 1.

**Table 1:** Comparison of participant characteristics between the case group and control group (Mean ± SD)

Participant characteristics	Case group (n = 30)	Control group (n = 30)	*P*-value
Age (year)	26.83 ± 3.58	26.93 ± 3.42	0.97
BMI	22.12 ± 1.43	22.02 ± 1.12	0.73
Pregnancy duration (day)	55.80 ± 5.85	52.57 ± 6.16	0.07
Number of previous pregnancies	2.43 ± 0.68	/	/

### Ethical approval and sample collection

The study was reviewed and approved by the Ethics Committee of Jiangxi Provincial Maternal and Child Health Hospital. All the participants signed the informed consent after proper explanation of the study. Blood samples were collected from each participant. In the case group, blood was collected 1 to 2 months after the abortion. Following centrifugation, the collected serum was stored at − 80 °C until proteomic analysis. In the control group, the sera of the 30 participants were divided into three samples, numbers 113, 114, and 115. In the case group, the sera of the 30 participants were also divided into three samples, numbers 116, 117, and 118.

### Protein extraction and peptide enzymatic hydrolysis

Serum pools were depleted of their most abundant proteins using Agilent Human 14/Mouse 3 Multiple Affinity Removal System Column following the manufacturer’s protocol [16–18] (Agilent Technologies). The supernatant was quantified with the BCA Protein Assay Kit (Bio-Rad, USA). Twenty micrograms of proteins in each sample were mixed with 5× loading buffer and boiled for 5 min. The proteins were separated on 12.5% SDS-PAGE gel (constant current 14 mA, 90 min). Protein bands were visualized with Coomassie Blue R-250 staining. A moderate amount of protein was extracted from each sample, and trypsin enzymatic hydrolysis was performed using the filter aided proteome preparation (FASP) method, then desalting enzymolysis peptides was performed using C18 Cartridge. Lyophilized peptides were dissolved with 40 μL dissolution buffer (OD280).

### iTRAQ labeling

One hundred micrograms of peptide mixture in each sample were labeled using iTRAQ reagent according to the manufacturer’s instructions (Applied Biosystems) [19].

### Peptide fractionation with strong cation exchange (SCX) chromatography

The iTRAQ labeled peptides were fractionated with SCX chromatography using the AKTA Purifier system (GE Healthcare). The dried peptide mixture was reconstituted and acidified with buffer A (10 mM KH_2_PO_4_ in 25% ACN, pH3.0) and loaded onto a Polysulfoethyl 4.6 × 100 mm column (5 μm, 200 Å, PolyLC Inc, Maryland, USA). The peptides were eluted at a flow rate of 1 ml/min with a gradient of 0–10% buffer B (500 mM KCl, 10 mM KH_2_PO_4_ in 25% ACN, pH3.0) for 25 min, 10–20% buffer B for 25–32 min, 20–45% buffer B for 32–42 min, 45–100% buffer B for 42–47 min, 100% buffer B for 47–60 min; buffer B was reset to 0% after 60 min. The elution was monitored with absorbance at 214 nm, and fractions were collected every 1 min. The collected fractions were desalted on C18 Cartridges (Empore™ SPE Cartridges C18 (standard density), bed I.D. 7 mm, volume 3 ml, Sigma), and concentrated with vacuum centrifugation.

### LC–MS/MS analysis

Each fraction was injected in the nano-LC–MS/MS for analysis. The peptide mixture was loaded onto a reverse phase trap column (Thermo Scientific Acclaim PepMap100, 100 μm * 2 cm, nanoViper C18) connected to the C18 reversed-phase analytical column (Thermo Scientific Easy Column, 10 cm long, 75 μm inner diameter, 3 μm resin) in buffer A (0.1% formic acid) and separated with a linear gradient of buffer B (84% acetonitrile and 0.1% formic acid) at a flow rate of 300 nl/min controlled with IntelliFlow technology. LC–MS/MS analysis was performed on a Q Exactive Mass spectrometer (Thermo Scientific) that was coupled to Easy nLC. The mass spectrometer was operated in positive ion mode. MS data were acquired using the data-dependent top 10 method that chooses the most abundant precursor ions from the survey scan (300–1800 m/z) for HCD fragmentation. Automatic gain control (AGC) target was set at 1e6, and maximum inject time to 10 ms. Dynamic exclusion duration was 40.0 s. Survey scans were acquired at a resolution of 70,000 at m/z 200, resolution for HCD spectra was set to 17,500 at m/z 200, and isolation width was 2 m/z. The normalized collision energy was 30 eV and the underfill ratio, which specifies the minimum percentage of the target value likely to reach the maximum fill time, was defined as 0.1%. The instrument was operating with the peptide recognition mode enabled.

MS/MS spectra were searched using the MASCOT engine (Matrix Science, London, UK; version 2.2) embedded into Proteome Discoverer 1.4 [20]. The protein screening criteria for identification were FDR less than 0.01, and the differentially expressed proteins were screened with multiple changes greater than 1.2 times (iTRAQ labeling) and a *P*-value less than 0.05.

### PRM

**Fig. 1:**
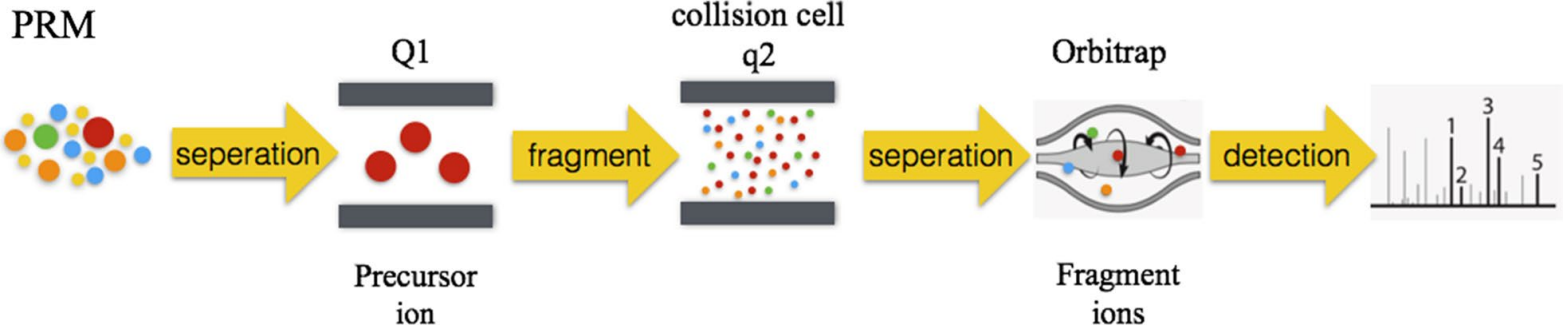
An experimental workflow for PRM method

Samples identified with the above mass spectrometry were verified by PRM. The experimental procedure was following (refer to Fig. 1). First, a PRM method is established in the original sample. The experiment is performed after the method is determined to be stable and reliable. We take the same number of peptides in each sample, and mix the appropriate amount of stable isotope internal standard peptide. We use the pre-experimental PRM method to detect the target protein in each sample using LC-PRM/MS. The results of PRM mass spectrometry were analyzed with Skyline quantitative analysis. After the internal standard peptide signal was corrected, the expression level of the target protein was obtained in each sample. The expression levels of the target proteins in different groups of samples were analyzed with student’s t-test.

### Statistics

Clinical data were expressed as mean ± standard error of the mean (S.E.M.). Statistical analysis was performed with SPSS software version 20.0 (SPSS, Inc., Chicago, IL, USA). Student’s t-test was applied for comparisons of quantitative data between the two groups, with *P* < 0.05 showing a significant difference. Multidimensional statistical test were calculated to estimate whether protein expression can predict the type of sample. Fisher’s exact test was used for categorical analysis. Data from the iTRAQ experiment and the original PRM test were stored at ProteomeXchange (http://proteomecentral.proteomexchange.org/cgi/GetDataset). Their IDs are 318751 and 318759, respectively.

## Results

### Differential protein identification results

**Fig. 2:**
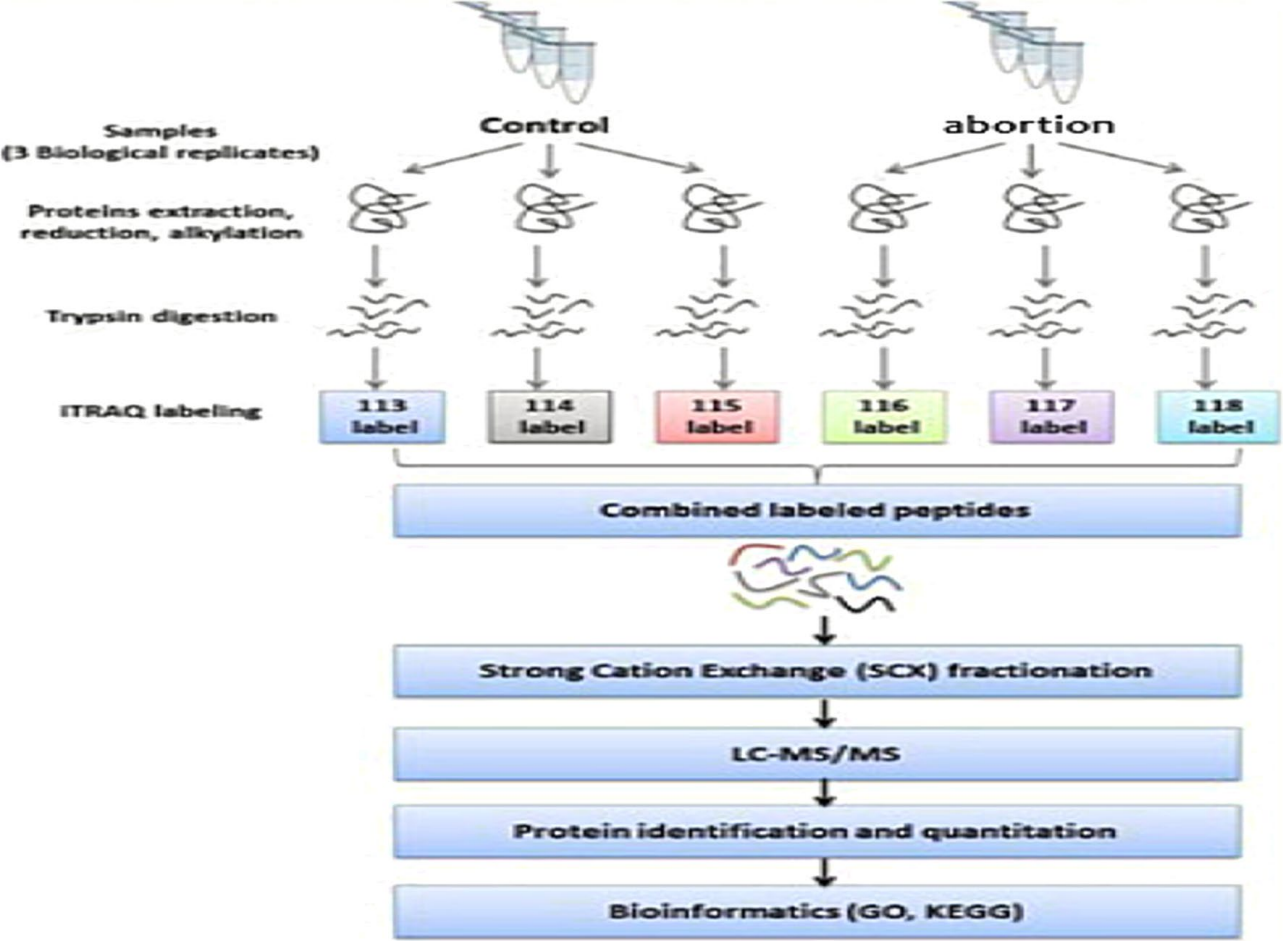
An experimental workflow for protein profiling of 30 patients with normal pregnancy and 30 patients with RSA, independently

In this project, differential proteomics analysis was performed in the serum of pregnant women with recurrent spontaneous abortion and normal pregnancy using the iTRAQ experimental method. The samples of the case group and the control group were labeled with n-label (adopting the 6-label method), and the differential proteomics detection was performed after labeling (Fig. 2).

A total of 977 proteins with unique peptides or polypeptide segments were identified, and a total of 40,855 characteristic peaks were identified (Table 2). Compared with the control group, in the case group, we found that 47 proteins were significantly down-regulated, while 31 proteins were significantly up-regulated (Table 3).

**Table 2:** Protein identification results

Database spectra (PSM)	Peak	Peptides	Unique peptides	Protein groups
Uniprot_Human_160426_20171024	40,855	6946	4380	977

**Table 3:** Differential protein expression profile

Accession	Description	Coverage	Unique peptide	Case/control	*P*-value
P02042	Hemoglobin subunit delta OS=Homo sapiens GN=HBD PE=1 SV=2-[HBD_HUMAN]	67.35	3	1.46	0.0495
P02743	Serum amyloid P-component OS=Homo sapiens GN=APCS PE=1 SV=2-[SAMP_HUMAN]	33.18	8	1.25	0.0258
E5RHP7	Carbonic anhydrase 1 (Fragment) OS=Homo sapiens GN=CA1 PE=1 SV=1-[E5RHP7_HUMAN]	21.12	5	1.52	0.0356
I1VZV6	Hemoglobin alpha 1 OS=Homo sapiens GN=HBA1 PE=3 SV=1-[I1VZV6_HUMAN]	57.75	1	1.47	0.0467
Q6MZL2	Putative uncharacterized protein DKFZp686M0562 (Fragment) OS=Homo sapiens GN=DKFZp686M0562 PE=2 SV=1-[Q6MZL2_HUMAN]	29.6	7	1.21	0.0306
B4DF70	cDNA FLJ60461, highly similar to Peroxiredoxin-2 (EC 1.11.1.15) OS=Homo sapiens PE=2 SV=1-[B4DF70_HUMAN]	42.62	6	1.29	0.0346
P27348	14-3-3 protein theta OS=Homo sapiens GN=YWHAQ PE=1 SV=1-[1433T_HUMAN]	15.92	1	1.21	0.0320
H3BMH2	Ras-related protein Rab-11A (Fragment) OS=Homo sapiens GN=RAB11A PE=4 SV=1-[H3BMH2_HUMAN]	20.65	3	1.27	0.0487
A0A087WZR4	Low affinity immunoglobulin gamma Fc region receptor III-B OS=Homo sapiens GN=FCGR3B PE=1 SV=1-[A0A087WZR4_HUMAN]	12.5	1	1.35	0.0412
A0A075B6R9	Protein IGKV2D-24 (Fragment) OS=Homo sapiens GN=IGKV2D-24 PE=4 SV=1-[A0A075B6R9_HUMAN]	10.83	1	1.48	0.0385
Q99650	Oncostatin-M-specific receptor subunit beta OS=Homo sapiens GN=OSMR PE=1 SV=1-[OSMR_HUMAN]	1.74	2	1.20	0.0148
P80188	Neutrophil gelatinase-associated lipocalin OS=Homo sapiens GN=LCN2 PE=1 SV=2-[NGAL_HUMAN]	19.7	3	1.21	0.0144
A8K061	cDNA FLJ77880, highly similar to Homo sapiens angiopoietin-like 3, mRNA OS=Homo sapiens PE=2 SV=1-[A8K061_HUMAN]	10.43	5	1.21	0.0379
P24593	Insulin-like growth factor-binding protein 5 OS=Homo sapiens GN=IGFBP5 PE=1 SV=1-[IBP5_HUMAN]	2.94	1	1.61	0.0375
Q9UBG0	C-type mannose receptor 2 OS=Homo sapiens GN=MRC2 PE=1 SV=2-[MRC2_HUMAN]	2.16	3	1.30	0.0276
Q9GZP0	Platelet-derived growth factor D OS=Homo sapiens GN=PDGFD PE=1 SV=1-[PDGFD_HUMAN]	3.24	1	1.24	0.0093
Q7Z2Y8	Interferon-induced very large GTPase 1 OS=Homo sapiens GN=GVINP1 PE=2 SV=2-[GVIN1_HUMAN]	0.66	1	2.29	0.0133
P35754	Glutaredoxin-1 OS=Homo sapiens GN=GLRX PE=1 SV=2-[GLRX1_HUMAN]	10.38	1	1.47	0.0214
H7C070	Uncharacterized protein KIAA1109 (Fragment) OS=Homo sapiens GN=KIAA1109 PE=1 SV=1-[H7C070_HUMAN]	0.37	1	1.32	0.0150
Q53FL1	Tumor endothelial marker 8 isoform 3 variant (Fragment) OS=Homo sapiens PE=2 SV=1-[Q53FL1_HUMAN]	3.79	1	1.20	0.0185
H0YD18	Nucleobindin-2 (Fragment) OS=Homo sapiens GN=NUCB2 PE=1 SV=2-[H0YD18_HUMAN]	12.33	1	1.20	0.0143
M0R266	IgG receptor FcRn large subunit p51 (Fragment) OS=Homo sapiens GN=FCGRT PE=1 SV=1-[M0R266_HUMAN]	4.96	1	1.37	0.0164
B7Z3I9	Delta-aminolevulinic acid dehydratase OS=Homo sapiens PE=2 SV=1-[B7Z3I9_HUMAN]	3.19	1	1.27	0.0271
Q5T619	Zinc finger protein 648 OS=Homo sapiens GN=ZNF648 PE=2 SV=1-[ZN648_HUMAN]	2.11	1	1.92	0.0407
B4E3Q1	cDNA FLJ61580, highly similar to Calsyntenin-1 OS=Homo sapiens PE=2 SV=1-[B4E3Q1_HUMAN]	1.35	2	1.33	0.0336
F6UYG0	Serine/threonine-protein kinase WNK1 OS=Homo sapiens GN=WNK1 PE=1 SV=1-[F6UYG0_HUMAN]	2.54	1	1.30	0.0051
A0A0J9YY48	Rab11 family-interacting protein 3 (Fragment) OS=Homo sapiens GN=RAB11FIP3 PE=4 SV=1-[A0A0J9YY48_HUMAN]	3.3	1	1.33	0.0384
P58166	Inhibin beta E chain OS=Homo sapiens GN=INHBE PE=1 SV=1-[INHBE_HUMAN]	2.29	1	1.32	0.0015
A0A024QZL1	Proteoglycan 1, secretory granule, isoform CRA_a OS=Homo sapiens GN=PRG1 PE=4 SV=1-[A0A024QZL1_HUMAN]	8.23	1	1.44	0.0119
B4E1S6	Syndecan OS=Homo sapiens GN=SDC4 PE=2 SV=1-[B4E1S6_HUMAN]	10.32	1	1.25	0.0010
C9J9F8	Coiled-coil domain-containing protein 173 (Fragment) OS=Homo sapiens GN=CCDC173 PE=4 SV=8-[C9J9F8_HUMAN]	3.8	1	1.71	0.00879
P01019	Angiotensinogen OS=Homo sapiens GN=AGT PE=1 SV=1-[ANGT_HUMAN]	43.71	1	0.68	0.0135
B4E1B3	cDNA FLJ53950, highly similar to Angiotensinogen OS=Homo sapiens PE=2 SV=1-[B4E1B3_HUMAN]	45.92	1	0.80	0.0144
B7Z8Q7	cDNA FLJ53871, highly similar to Inter-alpha-trypsin inhibitor heavy chain H4 OS=Homo sapiens PE=2 SV=1-[B7Z8Q7_HUMAN]	51.96	1	0.48	0.0001
B7Z9A0	cDNA FLJ56212, highly similar to Gelsolin OS=Homo sapiens PE=2 SV=1-[B7Z9A0_HUMAN]	45.43	1	0.71	0.0383
B3KS49	cDNA FLJ35478 fis, clone SMINT2007796, highly similar to Gelsolin OS=Homo sapiens PE=2 SV=1-[B3KS49_HUMAN]	51.55	1	0.56	0.0039
I3L145	Sex hormone-binding globulin OS=Homo sapiens GN=SHBG PE=1 SV=1-[I3L145_HUMAN]	75.58	17	0.82	0.0472
P0DOX2	Immunoglobulin alpha-2 heavy chain OS=Homo sapiens PE=1 SV=1-[IGA2_HUMAN]	46.15	5	0.71	0.0373
B2RBZ5	cDNA, FLJ95778, highly similar to Homo sapiens serpin peptidase inhibitor, clade A (alpha-1 antiproteinase, antitrypsin), member 10 (SERPINA10), mRNA OS=Homo sapiens PE=2 SV=1-[B2RBZ5_HUMAN]	40.54	1	0.80	0.0428
A0N5G3	Rheumatoid factor G9 light chain (Fragment) OS=Homo sapiens GN=V<lambda>3 PE=2 SV=1-[A0N5G3_HUMAN]	28.93	1	0.64	0.0082
A0A1B1CYC9	Vitamin D binding protein (Fragment) OS=Homo sapiens GN=Gc PE=4 SV=1-[A0A1B1CYC9_HUMAN]	40.63	6	0.47	0.0187
A0A0F7G8J1	Plasminogen OS=Homo sapiens GN=PLG PE=2 SV=1-[A0A0F7G8J1_HUMAN]	9.89	2	0.40	0.0005
P02675	Fibrinogen beta chain OS=Homo sapiens GN=FGB PE=1 SV=2-[FIBB_HUMAN]	19.55	7	0.80	0.0204
A8K430	Fructose-bisphosphate aldolase OS=Homo sapiens PE=2 SV=1-[A8K430_HUMAN]	23.35	6	0.71	0.0157
K7ELM3	Choriogonadotropin subunit beta variant 1 (Fragment) OS=Homo sapiens GN=CGB1 PE=3 SV=8-[K7ELM3_HUMAN]	49.61	5	0.24	0.0003
A0A0F7CSH9	Pregnancy-specific beta-1-glycoprotein 1 OS=Homo sapiens GN=PSG1 PE=2 SV=1-[A0A0F7CSH9_HUMAN]	14.11	2	0.45	0.0128
P11464	Pregnancy-specific beta-1-glycoprotein 1 OS=Homo sapiens GN=PSG1 PE=1 SV=1-[PSG1_HUMAN]	19.09	2	0.40	0.0212
A1L195	Tubulin beta chain (Fragment) OS=Homo sapiens GN=TUBB2B PE=2 SV=2-[A1L195_HUMAN]	22.87	2	0.73	0.0113
P01034	Cystatin-C OS=Homo sapiens GN=CST3 PE=1 SV=1-[CYTC_HUMAN]	15.75	3	0.58	0.0022
Q6P988	Palmitoleoyl-protein carboxylesterase NOTUM OS=Homo sapiens GN=NOTUM PE=1 SV=2-[NOTUM_HUMAN]	14.31	5	0.52	0.0017
P22692	Insulin-like growth factor-binding protein 4 OS=Homo sapiens GN=IGFBP4 PE=1 SV=2-[IBP4_HUMAN]	5.43	1	0.65	0.0094
B3KPT3	cDNA FLJ32147 fis, clone PLACE5000116, highly similar to Homo sapiens thrombospondin, type I, domain containing 3 (THSD3), transcript variant 1, mRNA OS=Homo sapiens PE=2 SV=1-[B3KPT3_HUMAN]	5.18	2	0.36	0.0040
B3KV07	cDNA FLJ16013 fis, clone PLACE5000171, highly similar to Mus musculus sushi, von Willebrand factor type A, EGF and pentraxin domain containing 1, mRNA OS=Homo sapiens PE=2 SV=1-[B3KV07_HUMAN]	2.88	2	0.56	0.0326
B4DTF1	cDNA FLJ51545, highly similar to Pregnancy-specific beta-1-glycoprotein 9 OS=Homo sapiens PE=2 SV=1-[B4DTF1_HUMAN]	20.16	4	0.39	0.0176
Q7Z7M8	UDP-GlcNAc:betaGal beta-1,3-N-acetylglucosaminyltransferase 8 OS=Homo sapiens GN=B3GNT8 PE=1 SV=1 - [B3GN8_HUMAN]	5.04	1	0.81	0.0187
B7Z6V5	cDNA FLJ50240, highly similar to ADAM DEC1 (EC 3.4.24.-) (Adisintegrin and metalloproteinase domain-like protein decysin 1) OS=Homo sapiens PE=2 SV=1-[B7Z6V5_HUMAN]	3.84	1	0.72	0.0169
B4DEU9	cDNA FLJ50120, highly similar to Homo sapiens mannosidase, alpha, class 2A, member 2 (MAN2A2), mRNA OS=Homo sapiens PE=2 SV=1-[B4DEU9_HUMAN]	5.17	2	0.62	0.0018
C9J6H2	Insulin-like growth factor-binding protein 1 OS=Homo sapiens GN=IGFBP1 PE=1 SV=1-[C9J6H2_HUMAN]	6.48	1	0.53	0.0016
A0A1U9X793	APOM OS=Homo sapiens PE=4 SV=1-[A0A1U9X793_HUMAN]	25	1	0.70	0.0413
Q96SB0	Anti-streptococcal/anti-myosin immunoglobulin lambda light chain variable region (Fragment) OS=Homo sapiens PE=2 SV=1-[Q96SB0_HUMAN]	37.96	2	0.57	0.0322
A0A0A0MT36	Protein IGKV6D-21 (Fragment) OS=Homo sapiens GN=IGKV6D-21 PE=4 SV=1-[A0A0A0MT36_HUMAN]	10.53	1	0.70	0.0042
Q5NKU1	Intercellular adhesion molecule 3 (Fragment) OS=Homo sapiens GN=ICAM3 PE=2 SV=1-[Q5NKU1_HUMAN]	3.53	2	0.74	0.0140
B7Z1C5	Glutathione synthetase OS=Homo sapiens PE=2 SV=1-[B7Z1C5_HUMAN]	5.43	2	0.63	0.0377
Q5JRP2	Disintegrin and metalloproteinase domain-containing protein 12 (Fragment) OS=Homo sapiens GN=ADAM12 PE=1 SV=1-[Q5JRP2_HUMAN]	3.6	1	0.43	0.0136
B7Z9E9	cDNA, FLJ78813, highly similar to Homo sapiens abl-interactor 1 (ABI1), transcript variant 3, mRNA OS=Homo sapiens PE=2 SV=1-[B7Z9E9_HUMAN]	15	2	0.75	0.0250
Q96IE3	Similar to plectin 1, intermediate filament binding protein, 500kD (Fragment) OS=Homo sapiens PE=2 SV=1-[Q96IE3_HUMAN]	2.14	2	0.70	0.0337
Q9Y646	Carboxypeptidase Q OS=Homo sapiens GN=CPQ PE=1 SV=1-[CBPQ_HUMAN]	3.6	2	0.65	0.0051
B4DEU8	cDNA FLJ60233, highly similar to Liprin-alpha-3 OS=Homo sapiens PE=2 SV=1-[B4DEU8_HUMAN]	1.39	1	0.42	0.0009
P18065	Insulin-like growth factor-binding protein 2 OS=Homo sapiens GN=IGFBP2 PE=1 SV=2-[IBP2_HUMAN]	6.46	2	0.76	0.0230
B7Z8Y6	cDNA FLJ58394, highly similar to Platelet endothelial cell adhesion molecule OS=Homo sapiens PE=2 SV=1-[B7Z8Y6_HUMAN]	3.41	2	0.82	0.0120
B4DND4	cDNA FLJ50588, highly similar to Gamma-glutamyltransferase 5 (EC 2.3.2.2) OS=Homo sapiens PE=2 SV=1-[B4DND4_HUMAN]	2.2	1	0.40	0.0432
Q8TER0	Sushi, nidogen and EGF-like domain-containing protein 1 OS=Homo sapiens GN=SNED1 PE=2 SV=2- [SNED1_HUMAN]	2.26	2	0.83	0.0155
B4E0T0	cDNA FLJ51589, highly similar to Neutrophil collagenase (EC 3.4.24.34) OS=Homo sapiens PE=2 SV=1-[B4E0T0_HUMAN]	4.26	1	0.59	0.0490
Q9HB00	Desmocollin 1, isoform CRA_b OS=Homo sapiens GN=DSC1 PE=4 SV=1 - [Q9HB00_HUMAN]	3.81	2	0.81	0.0314
A0A0B4J1R4	4-hydroxyphenylpyruvate dioxygenase OS=Homo sapiens GN=HPD PE=1 SV=1-[A0A0B4J1R4_HUMAN]	2.04	1	0.66	0.0341
P13727	Bone marrow proteoglycan OS=Homo sapiens GN=PRG2 PE=1 SV=2 - [PRG2_HUMAN]	4.95	1	0.43	0.0394
C9J3B7	Wiskott-Aldrich syndrome protein (Fragment) OS=Homo sapiens GN=WAS PE=1 SV=8-[C9J3B7_HUMAN	9.72	1	0.54	0.0010
A0A140TA77	Receptor-type tyrosine-protein phosphatase C (Fragment) OS=Homo sapiens GN=PTPRC PE=1 SV=1-[A0A140TA77_HUMAN]	2.41	1	0.74	0.0243

**Fig. 3:**
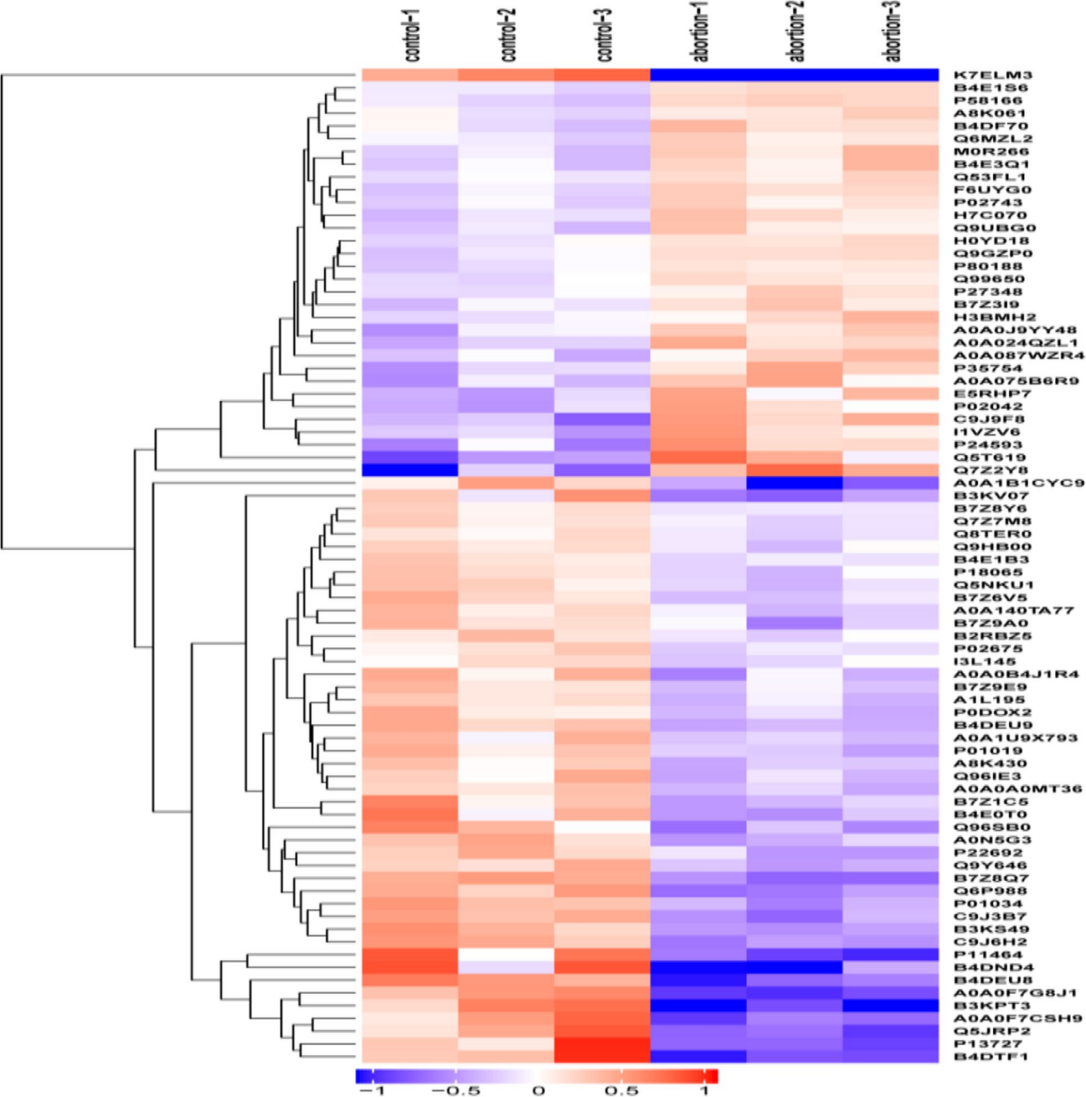
Cluster analysis of differentially expressed proteins in case_vs_control. Hierarchy clustering results expressed in tree heat maps, each row in the figure represents a protein, each column represents a set of samples, significant differences of protein level in the expression of different samples of numerical value (Log2Expression) to show different color in the heat map, the red represents significant increase protein, green represents significant lower protein, gray part represent quantitative information without protein

The differential protein in the clustering heat map (Fig. 3) can show that the biological repeats in the control and case group are good, and the protein level trends are consistent. Also, up- and down-changes are shown in the protein of both groups, and the screening standard was that multiple changes were greater than 1.2 (up-regulation greater than 1.2 or down-regulation less than 0.83) and the *P*-value was less than 0.05.

**Fig. 4:**
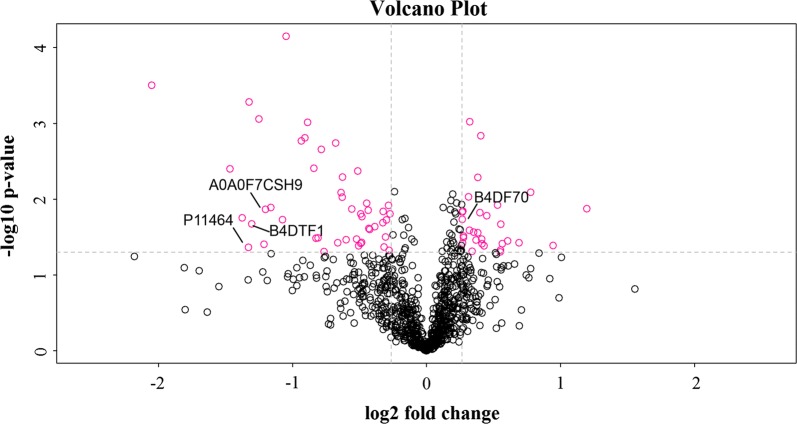
Case_vs_control group volcano plots. The fold change and the P-value obtained by T test were used to draw volcanic plots to show the significant differences between the two groups. Abscissa is the difference multiple (logarithmic transformation with base 2), ordinate is the significance of the difference, P-value (logarithmic transformation with base 10), red dots in the figure are the proteins with significant difference (P < 0.05), and black dots are the proteins with no difference

The differentially expressed proteins were visualized by mapping the volcano map (Fig. 4). Black represents non-differentiated protein, and red represents differentially expressed protein. The arrow indicates the PRM-validated proteins. In the figure, 4 target proteins are labeled, in which, B4DTF1, P11464, and B4DF70 were selected for PRM.

### Gene Ontology (GO) functional annotation and enrichment analysis of differentially expressed proteins

**Fig. 5:**
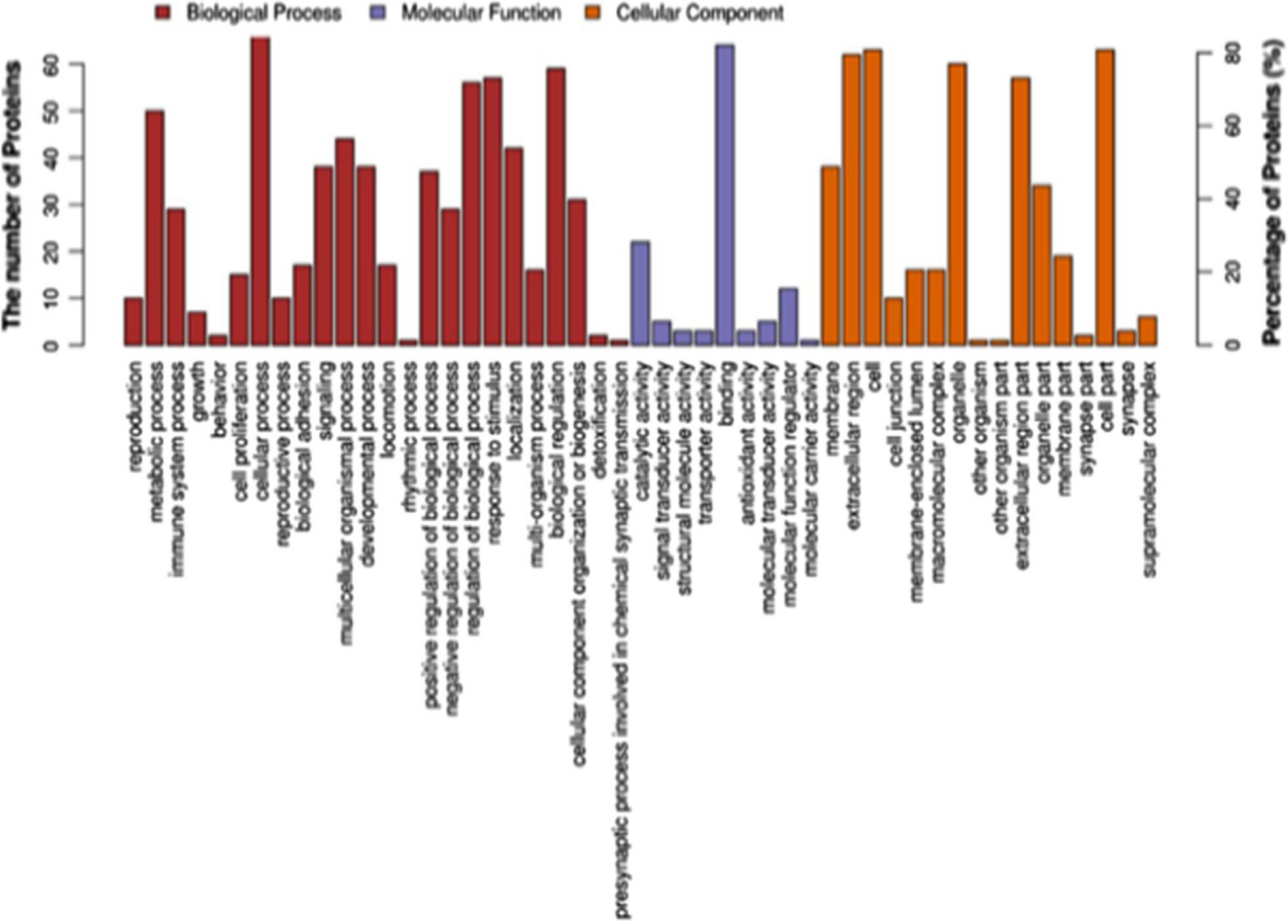
The GO annotation results of differentially expressed proteins in the case_vs_control group. The abscissa in the figure represents the GO Level 2 explanatory information, including biological process, molecular function and cellular component, which are distinguished by red, purple and orange respectively. The ordinate (right) represents the number of differentially expressed proteins under each functional classification, and the ordinate (left) represents the percentage of differentially expressed proteins under each functional classification in the total number of differentially expressed proteins

The differentially expressed proteins screened underwent GO function annotation using Blast2Go (https://www.blast2go.com/) software. Based on the results of the second level (Level 2), these differentially expressed proteins are primarily involved in cellular process, biological regulation, response to the stimulus, regulation of biological process, and metabolic process. The differentially expressed proteins might have some molecular functions, such as binding, catalytic activity, molecular function regulator, signal transducer activity, or molecular transducer activity (Fig. 5).

**Fig. 6:**
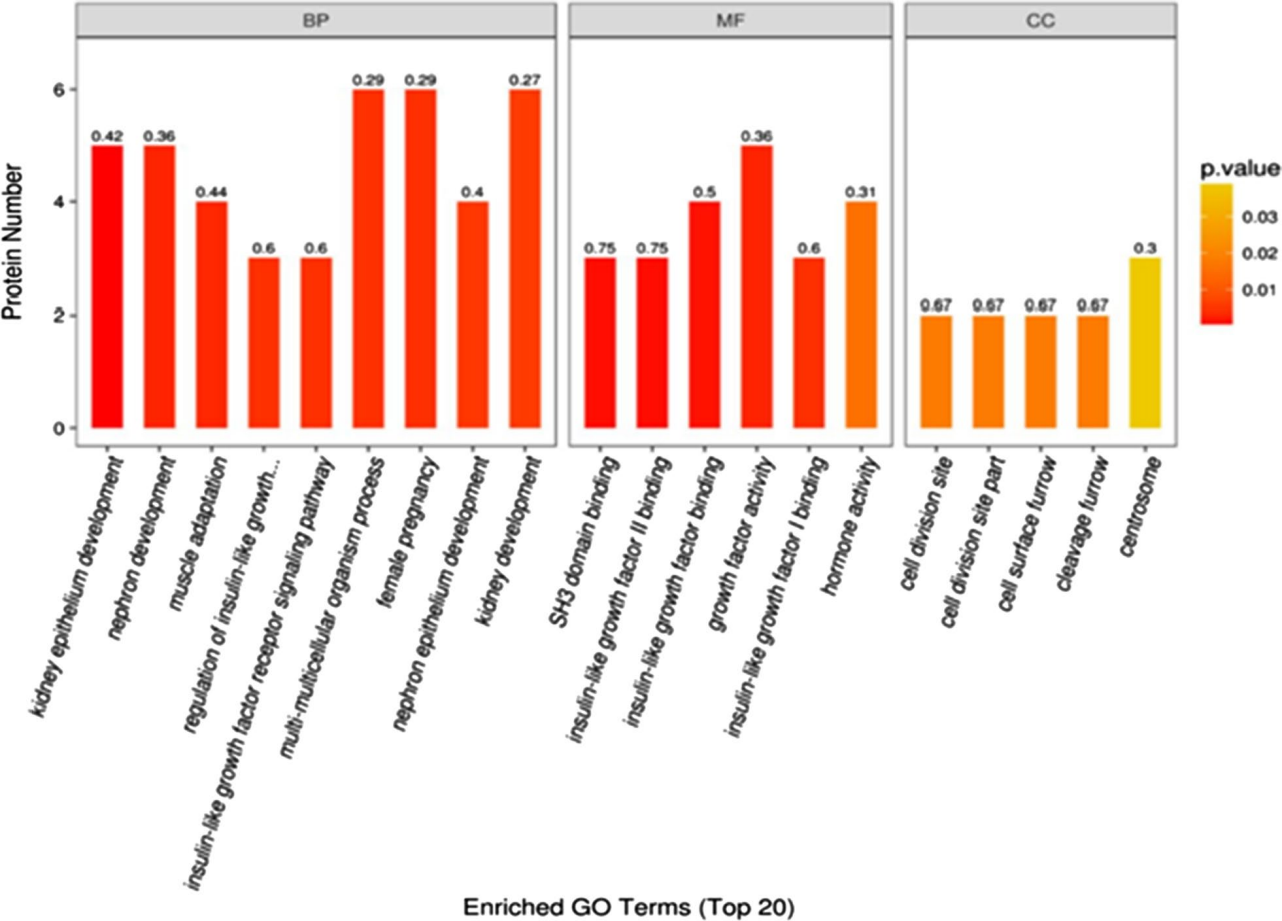
GO functional enrichment analysis in case_vs_control group. The abscissa in the graph show enrichment to GO function classification, which divided into a biological process (BP), molecular function (MF) and cellular components (CC) three categories; The ordinate represents the number of different proteins under each functional classification; The color of the bar chart represents the significance of enriched GO functional classification, that is, based on Fisher’s exact test to calculate the P value. The color gradient represents the size of P value. The color changes from orange to red. The label at the top of the bar chart shows the enrichment factor (richFactor ≤ 1), which represents the proportion of the number of differentially expressed proteins annotated into a GO function category to the number of all identified proteins annotated into the GO function category

As shown in Fig. 6, the GO functional enrichment analysis of differentially expressed proteins using Fisher’s exact test method, showed that these differential proteins were involved in critical biological processes, such as kidney epithelium development, nephron development, muscle adaptation, insulin-like growth factor receptor signaling pathway, or regulation of insulin-like growth factor receptor signaling pathway. Significant changes occurred in some molecular functions like SH3 domain binding, growth factor activity, insulin-like growth factor binding, and in localized proteins at cell division site part, cell surface furrow, cleavage furrow, cell division site, and centrosome.

### KEGG pathway annotation and enrichment analysis of differentially expressed proteins

**Fig. 7:**
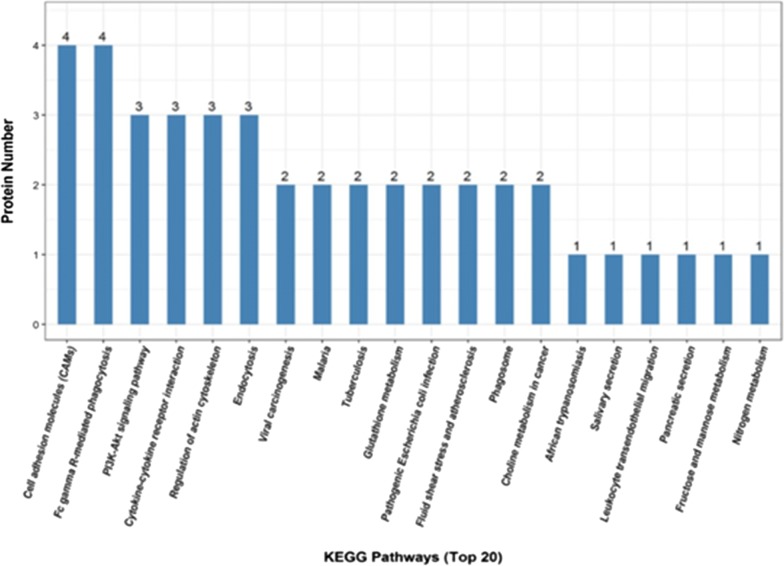
Case_vs_control group showed the results of the first 20 KEGG pathways with the most differentially expressed proteins. The abscissa in the figure is the name of the pathway in which the differentially expressed proteins are involved, and the ordinate is the number of differentially expressed proteins involved in the pathway. In general, the higher the number of differentially expressed proteins corresponds to a certain pathway, the more important the pathway is

KEGG pathway analysis indicated that differentially expressed proteins are located in important pathways such as cell adhesion molecules, Fc gamma R-mediated phagocytosis, PI3K–Akt signaling pathway cytokines, cytokine receptor interactions, and regulation of actin cytoskeleton (Fig. 7).

**Fig. 8:**
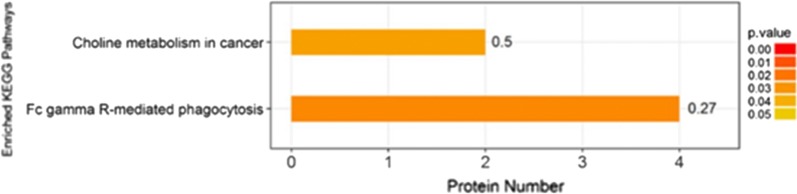
KEGG pathway enrichment analysis in case_vs_control group. The ordinate in the figure represents significantly enriched KEGG pathways. The abscissa represents the number of differentially expressed proteins contained in each KEGG pathway. As shown in the bar graph, color represents the significance of enriched KEGG pathways. Fisher’s exact test is used to calculate the *p*-value. Color gradient represents the size of *P*-value. The label at the top of the bar chart shows enrichment factor (richFactor ≤ 1), which represents the proportion of the number of differentially expressed proteins involved in a KEGG pathway to the number of proteins involved in this pathway among all identified proteins

KEGG pathway enrichment analysis of differentially expressed proteins with Fisher’s exact test revealed that significant changes have occurred in some important pathways, such as Fc gamma R-mediated phagocytosis, choline metabolism in cancer, taurine and cell adhesion molecules (CAMs), etc. (Fig. 8).

For example, in the Fc gamma R-mediated phagocytosis, the target proteins with noticeable differences include PTPRC (CD45), Gelsolin and WASP.

### Protein–protein interaction (PPI) network analysis

**Fig. 9:**
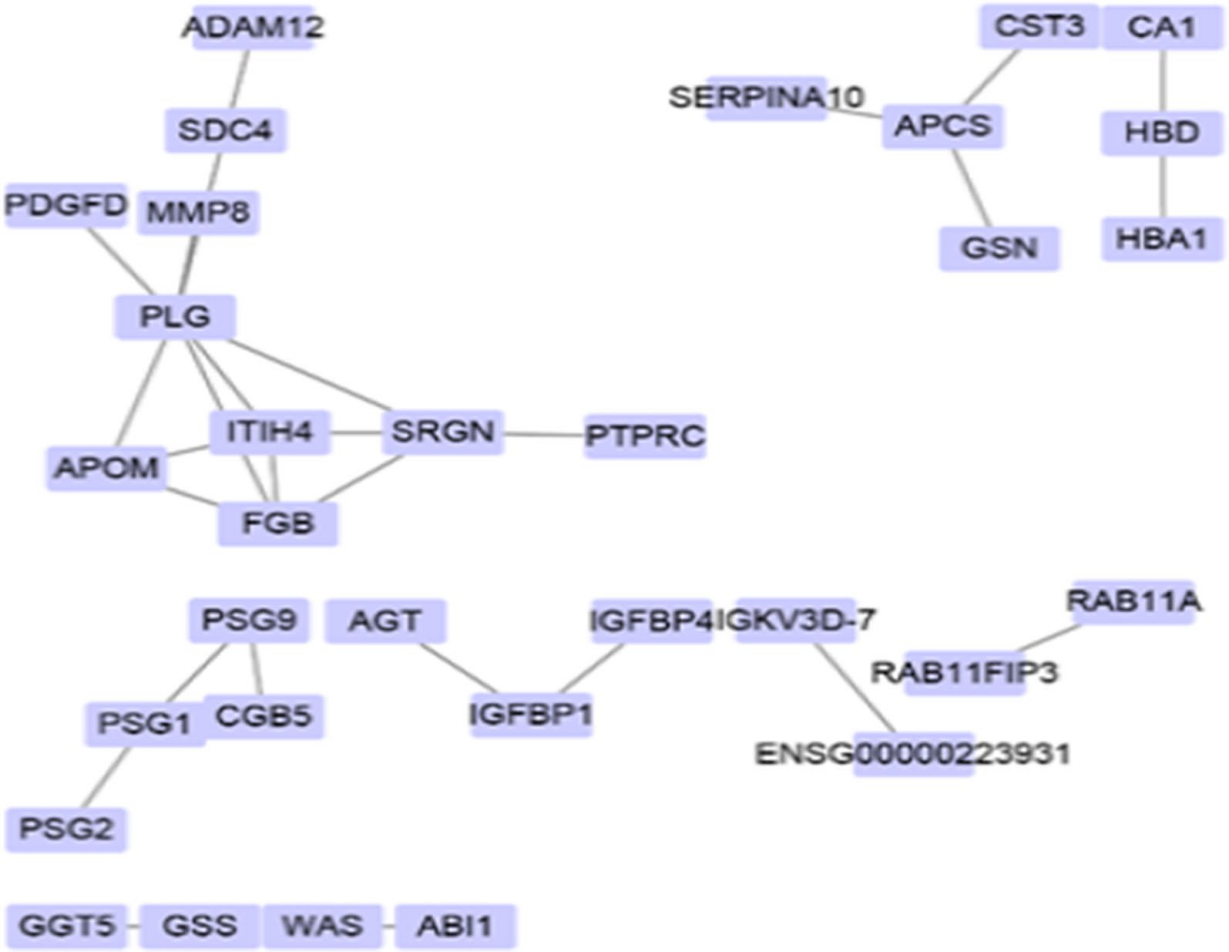
Interaction network of differentially expressed proteins in case_vs_control group. In the protein interaction network, nodes represent proteins and lines represent the interactions between proteins

The study of the interaction between proteins and the interaction network is of great significance for revealing the function of proteins. In a network, the number of proteins that interact directly with a protein is called the connectivity of that protein. In general, the greater the connectivity of the protein, the greater the disturbance in the whole system when the protein changes. The protein might be the key to maintain the balance and stability of the system, and it is a candidate protein for subsequent studies. By comparing proteins to STRING, the results showed that known proteins, such as ITIH4, PSGs, PLG, IFGBPs, FGB, APCS and CD45, account for a large weight in the network (Fig. 9).

### PRM results

Four proteins related to abortion were found for PRM analysis. In the experiment, PRM quantitative analysis was performed on five peptides of three target proteins in 12 human serum samples, and quantitative information of the target peptides was found in all the 12 samples. The isotope re-labeled peptides were used to normalize the quantitative information, and then the relatively quantitative analysis of the target peptides and target proteins was performed. The results of differential multiples and T-test showed that there were some differences in the levels of the three target proteins under these two different conditions, which was consistent with the results of omics verification. The results showed that B4DTF1and P11464 level was down-regulated, while B4DF70 level was up-regulated (Table 4). B4DTF1 and P11464 have similar efficacy, and they are similar to the function of Pregnancy-specific beta-1-glycoprotein 1 (PSG1), while B4DF70 are similar to Peroxiredoxin-2 (Prdx2), which might be associated with oxidative damage.

**Table 4:** Results of relatively quantitative analysis of target peptide

Protein name	Control_average	Abortion_average	Ratio_Abortion/control	TTEST_Abortion/control
B4DTF1	0.2775	0.0518	0.1867	0.010341499
P11464	0.5713	0.1188	0.2080	0.000123079
B4DF70	0.1781	0.2188	1.2284	0.412413567

## Discussion

The use of proteomics to identify key proteins associated with abortion can provide insight into the mechanisms of ERSA. In this study, we found 78 differentially expressed proteins using iTRAQ, and bioinformatics analysis demonstrated that these proteins were implicated in several biological processes and molecular functions. Six proteins were significantly different and they were consistent with PSG1, Prdx2, CD45, ITI-H4, IGFBP and INHBE, compared with the database. A total of three proteins were selected for PRM.

ITI-H4 and IGFBP have been reported to be associated with miscarriage [12, 13], findings similar to our results. INHBE was identified as a novel putative hepatokine with hepatic gene expression that positively correlated with insulin resistance and body mass index in humans [21]. Other similar studies showed that many differentially expressed proteins were identified, which were different from our results. For example, in 2014, Ni et al. [22] extracted all the proteins in the placental villus tissue of 5 patients with early spontaneous abortion and 5 patients with normal pregnancy requiring therapeutic abortion. Fifty-one differentially expressed proteins were identified using HPLC–MS. Bioinformatics analysis of the 12 proteins in these differential proteins might be involved in the biological process of spontaneous abortion, NES, P4HA2, PBXIP1 and GSTM2 are involved in the ability of trophoblasts to infiltrate the endometrium. The difference might be related to different proteomics techniques, different specimen and selected cases.

Moreover, our results indicated that the Fc gamma R-mediated phagocytosis might play an essential role in the mechanism of ERSA. CD45 were significantly down-regulated in this pathway. Similar to this result, Lorenzi et al. [23] found that fetal CD100, CD72 and CD45 were expressed in placenta and exhibited different mRNA and protein levels in normal pregnancy and miscarriage, CD45 was down-regulated in miscarriage.

According to PRM, PSG1 and Prdx2 were considered biomarkers of ERSA. In the field of obstetrics, especially in abortion, there are more reports on PSG1, but few reports on Prdx2.

Human PSGs were detected in the maternal serum after fertilized eggs have been implanted for 3 days, consistent with the time when the blastocyst adhered to the uterine wall [24]. PSGs are abundant in maternal serum, can induce the transformation of growth factor TGFβ-1, inhibit the function of T-cell, and promote angiogenesis [25]. It is important to have a better understanding of the molecules that control angiogenesis and trophoblast-mediated vascular remodeling during pregnancy. Because disorders of blood flow and vascular development in the placenta could affect fetal growth [26]. Angiogenesis occurs at various stages of pregnancy to ensure that the embryo receives adequate nutrients and oxygen [27, 28]. First, PSG1 can induce TGFβ-1 and VEGFA through different cell types, including monocytes, macrophages and natural killer cells [29, 30]. Second, PSG1 has the ability to interact with endothelial cells, induce angiogenesis, and enhance angiogenic processes [31–33]. To the best of our knowledge, the association between PSG1 and RSA has not been reported previously. PSG1 was originally described in the early 1970s, but more research will likely contribute to demonstrate their importance for a successful pregnancy [34].

Prdx2 is an antioxidant protein that uses its redox-sensitive cysteine group to reduce hydrogen peroxide molecules and protect cells from oxidative damage from reactive oxygen species (ROS). Its role in the maternal–fetal interface trophoblast has not been elucidated. A current study has shown that the expression of Prdx2 in the trophoblast of the patients with RSA within the first 3 months of pregnancy is significantly lower than in the healthy control group [35]. Applying Proteomic technology, a 2D-PAGE and MALDI-TOFMS study showed that Prdx2 was down-regulated in placental trophoblasts from patients with preeclampsia [36, 37]. However in our study, we found that the level of Prdx2 was up-regulated in the case group. We considered that the difference could be related to the difference of specimens.

During our study, we faced with some limitations. For example, the sample size was relatively small, and assessment consistency between the level of target protein with the expression in decidual tissues was not discussed.

## Conclusions

In conclusion, the present study used iTRAQ and PRM-based quantitative proteomics to find three biomarkers of ERSA. Compared with other similar studies, this study show improvement in detection techniques. This method can be more effective and accurate in the investigation of alterations in protein profiles. Furthermore, we identified PSG1, Prdx2, and CD45 as new serum biomarkers of ERSA, and their potential application in the maternal–fetal interface will require further study. Larger‑scale studies will be required to confirm the diagnostic value of these markers.

## Data Availability

All data generated or analyzed during this study are included in this published article.

## Abbreviations

2D-PAGE: two-dimensional polyacrylamide gel electrophoresis
CD45: protein tyrosine phosphatase receptor type C
ERSA: early recurrent spontaneous abortion
FASP: filter aided proteome preparation
GO: Gene Ontology
IGFBP: insulin‑like growth factor‑binding protein
INHBE: inhibin beta E
IPA: ingenuity pathway analysis
ITI-H4: inter-α trypsin inhibitor-heavy chain 4
iTRAQ: isobaric tags for relative and absolute quantification
KEGG: Kyoto Encyclopedia of Genes and Genomes
LC: liquid chromatography
MALDI-TOF: matrix-assisted laser desorption–ionization time of flight
MS: mass spectrometry
PPI: protein–protein interaction
Prdx-2: peroxiredoxin-2
PRM: parallel reaction monitoring
PSG1: pregnancy-specific beta-1-glycoprotein 1
RAGE: receptor for advanced glycation end products
RPL: recurrent pregnancy loss
ROS: reactive oxygen species
SCX: strong cation exchange
